# Electrospray patterning of yeast cells for applications in alcoholic fermentation

**DOI:** 10.1038/s41598-019-55225-4

**Published:** 2019-12-09

**Authors:** Sang Bin Jeong, Eui-seok Chong, Ki Joon Heo, Gun Woong Lee, Hyung Joo Kim, Byung Uk Lee

**Affiliations:** 1 Aerosol and Bioengineering Laboratory, Department of Mechanical Engineering, Konkuk University, 120 Neungdong-ro, Gwangjin-gu, Seoul 05029 Republic of Korea; 2 Department of Biological Engineering, Konkuk University, 120 Neungdong-ro, Gwangjin-gu, Seoul 05029 Republic of Korea

**Keywords:** Biotechnology, Engineering, Nanoscience and technology

## Abstract

Yeast cells patterned by pulsed jet electrospray showed a high alcoholic fermentation rate. Multi-dimensional patterns of individual yeast cells were produced by varying the experimental parameters of the electrospray system. The electrospray process, which employed a vibrational electric field, could control patterns of viable yeast cells at a cellular resolution. This novel system for electrospraying viable cells can be applied to biological process engineering including whole cell biochip techniques and micro fermentation processes for biochemical studies.

## Introduction

The immobilization of cells on solid surfaces has been applied in the development of bioreactors, biochemical tests, and *in vitro* pharmacokinetics. Microenvironments on solid surfaces enhance cell stability, cell recycling, and downstream cellular processes^1^. Microcontact printing, photolithography, dip-pen nanolithography, and inkjet printing are considered as methods for patterning biomaterials on substrates^2–6^. However, these patterning methods have drawbacks as they can be time-consuming (microcontact printing and photolithography), induce chemical damages on cells (dip-pen nanolithography), or be inaccurate in controlling the position of individual cells (microcontact printing, photolithography, and inkjet printing)^6,7^. An electrospray method developed for the atomization of fine droplet particles^8–12^ has been applied for the accurate analysis of biological particles^13–16^. As a new application for the electrospraying technique, cell patterning using an electrospray pulsed jet was developed^17,18^, and this method allowed accurate immobilization of individual cells on an untreated substrate without chemical damage^19^. Previous studies presented that non-agglomerated cells that were electrically charged via pulsed electrical fields between a nozzle and a ground plate in the electrospray system showed patterns at a cellular resolution^18,20–22^. In this study, an electrospray patterning system for yeast cells was created. The patterns of individual yeast cells were fabricated at a cellular resolution, and the electrospray-patterned cells showed high-speed alcoholic fermentation rates.

## Results

### Electrospray patterning of live yeast cells

**Figure 1:**
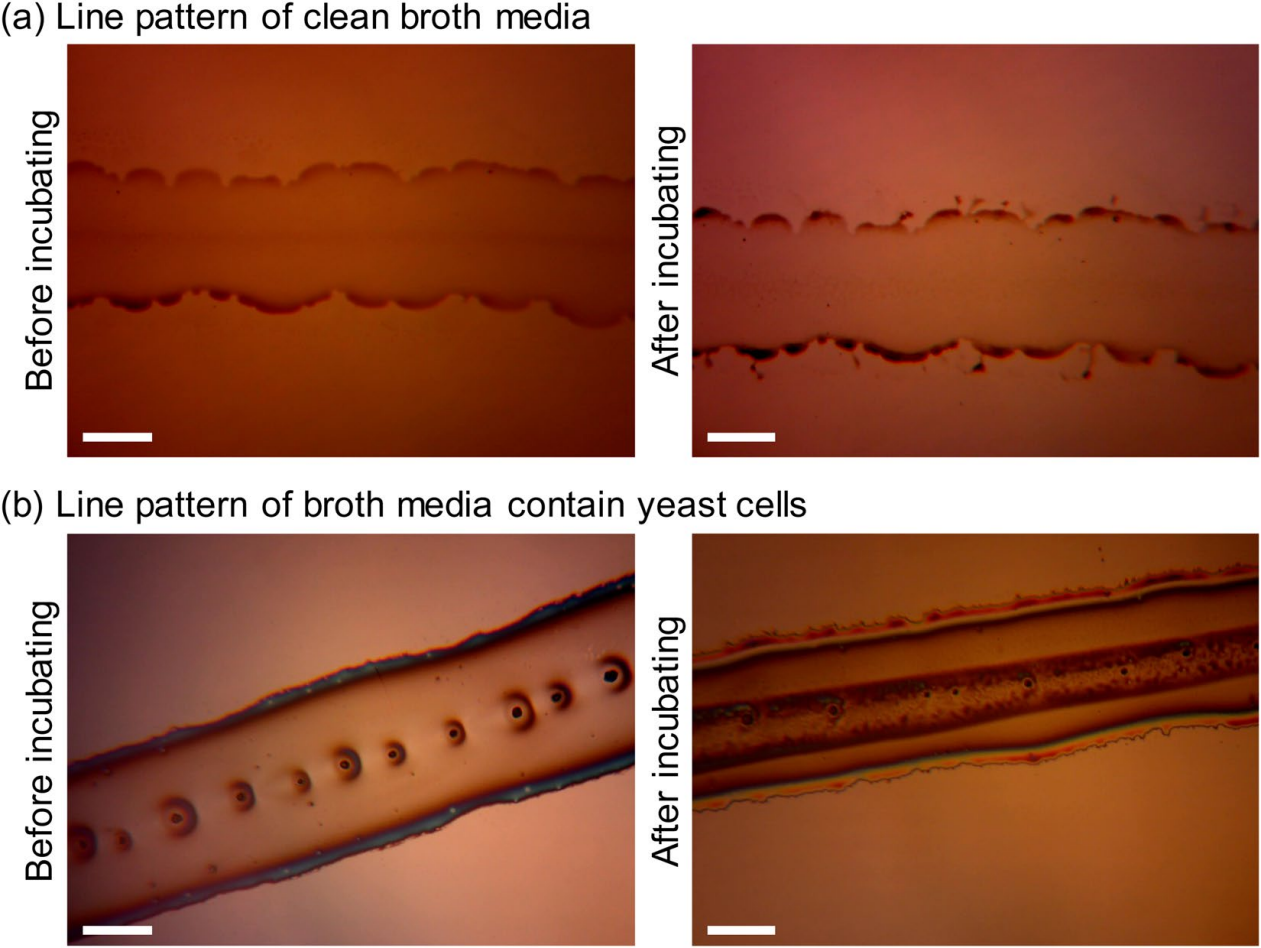
Electrospray line pattern of broth medium and yeast cell (*Saccharomyces cerevisiae*) containing medium. (**a**) Line pattern of broth medium without yeast cells. (**b**) Line pattern of yeast cell-containing medium. Incubation was conducted at 25 °C for 24 h after the deposition process. Scale bars represent 100 μm.

We generated line patterns of materials at a translation speed of 1 mm/s under a vibrating electrical field of 0.8 kV and at a frequency of 20 Hz. Nutrient broth media were patterned at a flow rate of 5 µL/h and incubated at 25 °C for 24 h. The line patterns of clean nutrient broth medium (Becton Dickinson, Franklin Lakes, NJ, USA) (Fig. 1a) had an average width of 191 µm, and the broth medium patterns before and after incubation did not show significant differences. Aside from the evaporation on the edges, the broth medium mostly maintained its shape after incubation as showed in Fig. 1a. Figure 1b demonstrates the yeast cell patterns before and after incubation. The line patterns of broth medium containing yeast cells (Fig. 1b) had an average width of 252 µm. In addition, Fig. 1b shows that the patterned yeast medium generated a darker line in the middle with an average width of 79 µm after incubation. This dark line seems to be the extracellular matrix (ECM) that acts as a cell-supporting scaffold^23^, indicating that the yeast cell viability did not vanish even after the cells passed through the electrospray patterning system. The viability of the patterned yeast cells was also confirmed by an increase in the number of incubated yeast cells (Fig. 1b).

### One-dimensional drop-on-demand patterning of yeast cells

**Figure 2:**
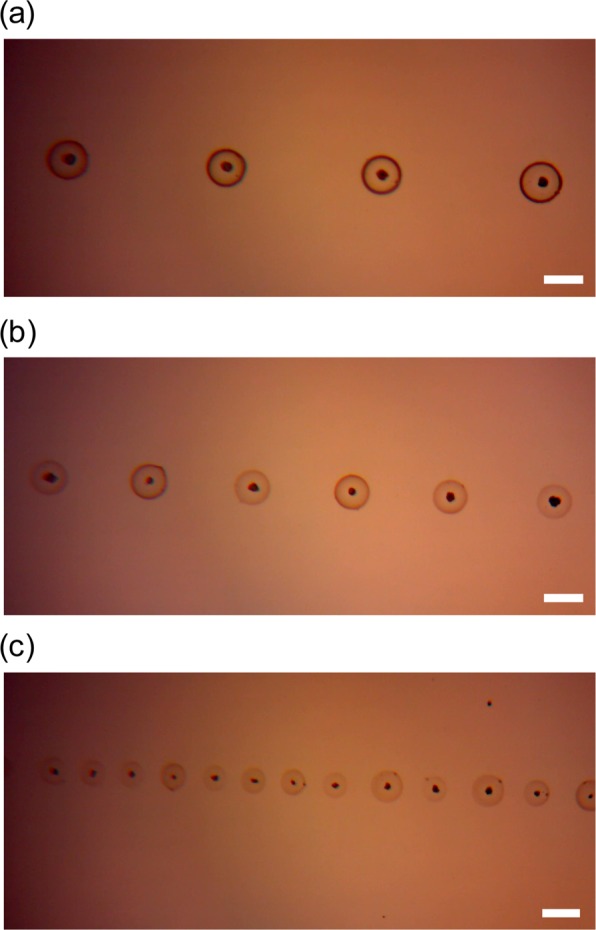
One-dimensional patterned yeast cell medium. The average distances between the center points of two droplets were (**a**) 158 μm at a translation speed of 1.5 mm/s, (**b**) 83 μm at a translation speed of 1 mm/s, and (**c**) 30 μm at a translation speed of 0.5 mm/s. Scale bars represent 50 μm.

Figure 2a shows the drop-on-demand patterning of yeast cells, which consisted of spots with a regular diameter of 50 µm and an average inter-spot distance of 158 µm. Individual spots contained one yeast cell or very few cells, indicating that the aggregating nature of yeast cells could be controlled by electric forces in the electrospray system. The distance between the droplets could be varied by adjusting translation speed, which ranged from 0.5 to 1.5 mm/s. As the translation speed decreased, the distance between two droplets also decreased. The inter-spot distance was 83 μm at a translation speed of 1 mm/s (Fig. 2b) and decreased to 30 μm at a speed of 0.5 mm/s (Fig. 2c). In addition, the droplet size and number of cells in a single droplet decreased at higher translation speeds.

**Figure 3:**
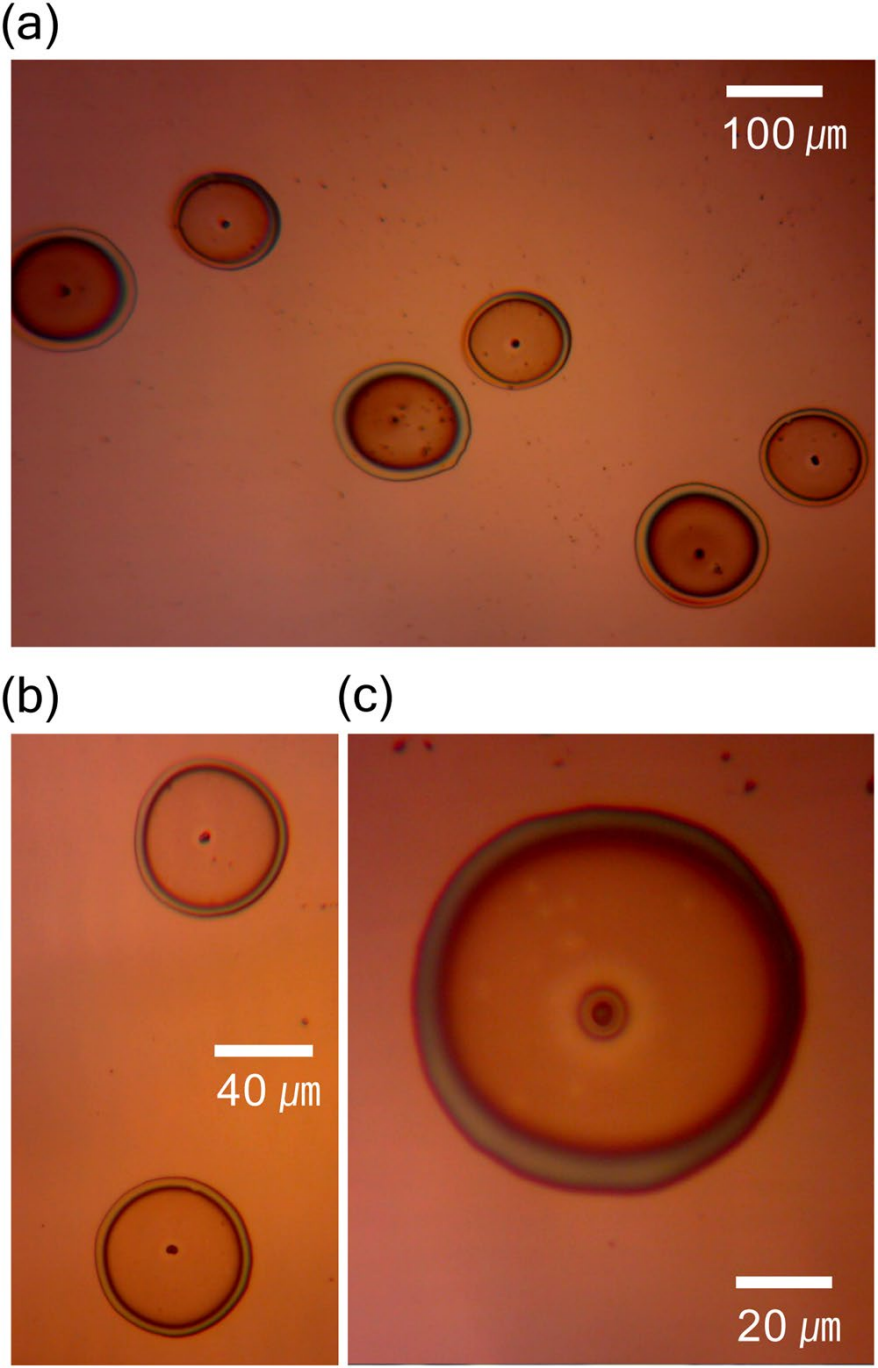
Magnified photographs of yeast cells patterned using the electrospray system. (**a**) Six patterned yeast cells. (**b**) Two patterned yeast cells (yeast cells located at the center of the individual spots). (**c**) Single yeast cell at the center of the droplet.

The high-resolution images of yeast patterns (Fig. 3) demonstrate that the droplet diameters varied with the flow rates. Small droplets were produced at a flow rate of 4 μL/h and large droplets were produced at 5 μL/h (Fig. 3a). The experimental conditions (translation speed of 2 mm/s, alternating-current (AC) electricity at 0.8 kV, and frequency of 20 Hz) were identical in these two cases. The geometric mean diameters of the small and large droplets were 103 µm and 128 µm, respectively. Single yeast cells with a diameter of 3 μm and an elliptical shape were patterned in individual spots, as shown in Fig. 3b,c. In addition, the patterned yeast cells tended to localize at the center of each droplet. The cone-jet electrospraying mode generated highly electrically charged monodisperse droplets and particles. The electrostatic repulsive forces of these particles may cause them to repel each other. Therefore, individual yeast cells in the droplets may be localized in the center of the spots because of the balance among the electrostatic repulsive forces^24,25^.

### Fermentation efficiency of electrospray-patterned yeast cells

In this experiment, alcoholic fermentation by the patterned yeast cells was performed using a solution of squeezed grapes. Alcohol concentration was measured over 120 h in all individual tests and served as an indicator of fermentation in the solution. Squeezed grape solutions without yeast cells were used as control materials for the experiment. We hypothesized that the fermentation efficiency was correlated with how yeast cells were spread in the grape solution under the same cell number conditions. To verify this hypothesis, we conducted experiments with yeast cells under various conditions: dropping the yeast medium using a pipette (“No-patterned”); using an air pressure unit (“fine-mist sprayer”); and positioning yeast cells inside the solution with electrospray droplet patterns of 30 μm, 100 μm, or 1,000 μm (“patterned”).

**Figure 4:**
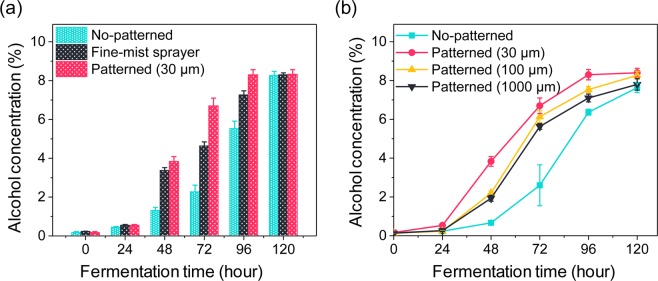
Alcohol concentrations during the fermentation processes. (**a**) Fermentation conditions: dropping the yeast medium using a pipette (“No-patterned”); using an air pressure unit (“fine-mist sprayer”); and positioning yeast cells inside the solution with electrospray patterns of 30 μm (“patterned”). (**b**) Fermentation conditions: (“No-patterned”) and (“patterned”) with 30 μm, 100 μm, and 1,000 μm droplet patterns. Error bars indicate standard deviations (n = 3).

Figure 4 shows the experimental fermentation results. The alcohol concentration in the control solution (incubation without yeast cells) ranged from 0.1% to 0.3%. Yeast cells artificially mixed in the grape solution induced the fermentation process. The alcohol concentration in the yeast solutions converged from 8% to 8.4% after 120 h of fermentation. However, the fermentation rate varied significantly depending on the type of yeast cell spreading in the grape solution. Yeast cells patterned at 30 μm showed the highest fermentation rate, whereas those without patterns presented the lowest rate. The fermentation rate increased from 0.08% per hour to 0.1% per hour by varying yeast cell spreading from the non-patterned to the patterned form (at 30 μm). As shown in Fig. 4, the 30 μm pattern showed the highest fermentation efficiency at all time points. Furthermore, the 30 μm pattern decreased the time for alcohol production via yeast cell fermentation by 25%. This result demonstrates that yeast cell patterning can be applied to increase the alcohol production rate by fermentation.

Figure 2c shows the small droplets that were produced by patterning the cells at 30 μm. As the droplet size decreased, contact between the squeezed grape solution and yeast cells was promoted, which was expected to increase the amounts of the fermentation reactions on the droplet surfaces. The effect of specific electrospray operating conditions such as flow rate, frequency, and voltage on yeast fermentation efficiency could be explored in future studies on yeast cell electrospray patterning systems. Our study focused on the fermentation potential of viable electrospray-patterned yeast cells, and we did not explore the usage of the produced alcohol in products such as fuel or beverages. In the future, to apply this new technology for the production of such commodities, its effect on flavor and production speed would need to be tested.

## Conclusions

A new electrospray drop-on-demand patterning system was created to pattern viable yeast cells at a cellular resolution. Viable electrospray-patterned yeast cells showed high-speed alcoholic fermentation rates. In this experiment, the 30 μm droplet pattern of yeast cells showed the most efficient performance in alcohol fermentation. This electrospray cell patterning technology can be applied to biochemical engineering and fermentation industries.

## Methods

**Figure 5:**
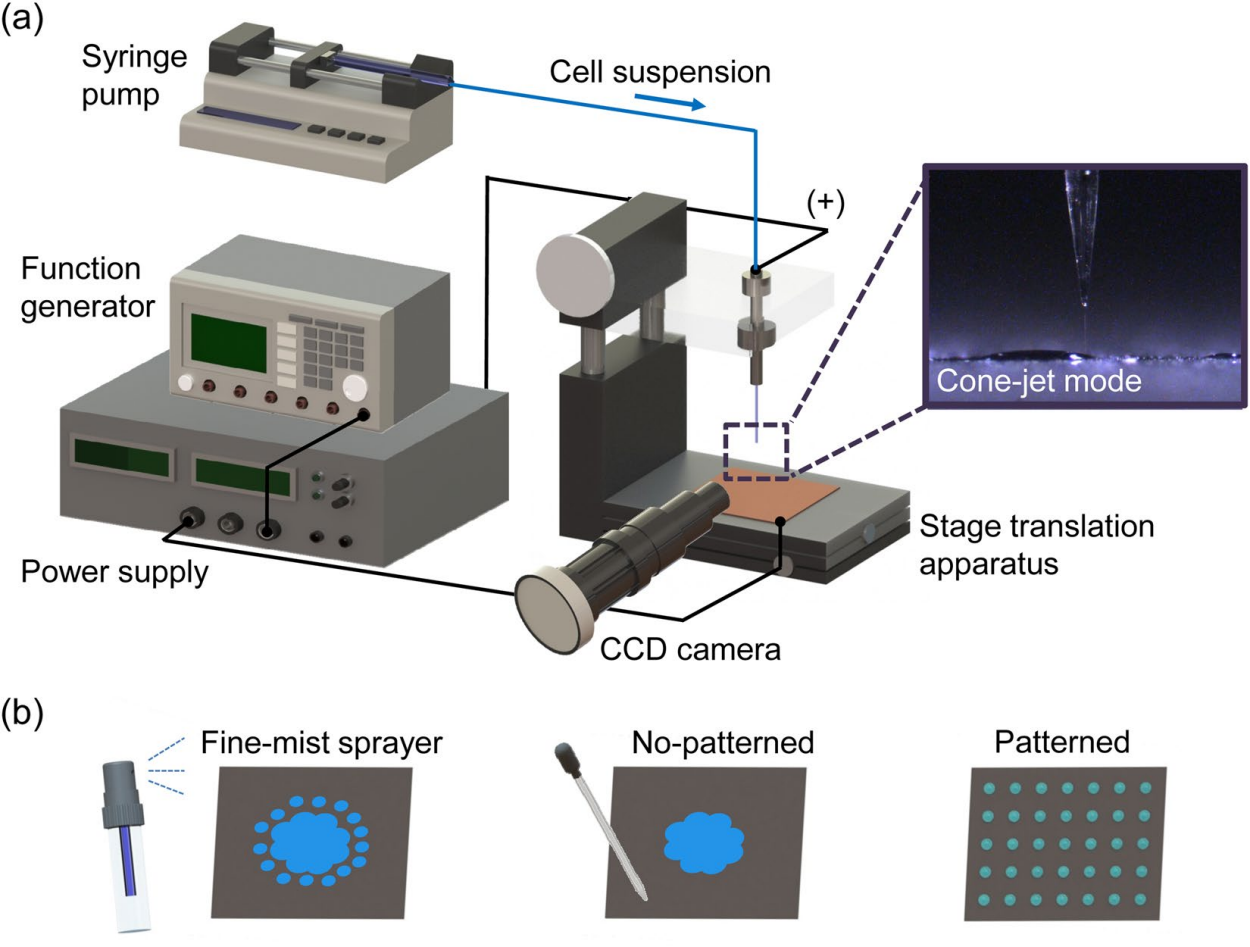
(**a**) Schematic diagram of the electrospray patterning system for yeast cells. The stable cone-jet mode and two-way stage translation apparatus enabled patterning of living cells. (**b**) Various yeast cell spreading conditions: spreading using a fine-mist sprayer, non-patterned spreading, and patterned spreading with cell distances of 30, 100, and 1,000 μm.

Figure 5a shows the experimental configuration of the electrospray patterning system, which was created based on our previous study^18^. The experimental setup consisted of a liquid supply system with a capillary tube, a high-voltage power supply system, a ground plate with a translation system, and a visualization system. A yeast suspension was loaded into silica capillaries (PicoTip emitter, New Objective, USA) with a diameter of 30 μm using a 25 μL syringe (1702TLL, Hamilton, USA) and a syringe pump (Model 220, KD Scientific, USA). A power supply device (DC-AC 15 kV, Korea Switching, Korea) with a function generator (FG-7002C, EZ Digital, Korea) was used to generate an electric field between the silica capillaries and the ground plate. Electricity was applied to a stainless steel ZDV union (U-322, Upchurch, USA) to confer charge to the passing yeast cell suspension during electrospraying. A silicon wafer with a diameter of 12 cm placed on a stainless steel plate was used as a ground plate for the electrospray system. A two-dimensional stage translation apparatus with adjustable constant speed was used to produce patterns of suspension particles in a vibrating electrical field between the capillary tip and the ground plate installed 1 mm below. The visualization system shown in Fig. 5a, which consisted of a light source (LS-100W, Light Solution, Korea) and a charge coupled device camera (Marlin F-145C2, Allied Vision Tech, Germany) with a zoom lens (70XL, OPTEM, Korea), was employed to monitor the electrospray mode in real time. In addition, the yeast particle patterns produced on the wafer were observed by an optical microscope (Eclipse ME600, Nikon, Japan) equipped with a charge-coupled device camera (INFINITY1, Lumenera Co., Canada).

For the drop-on-demand patterning experiments, electricity was supplied at 0.8 kV with a frequency of 20 Hz by a high-voltage power supply system. We applied vibrating sinusoidal high-voltage electricity at several kilovolts to the capillary by means of a high-voltage AC power supply (AC +15 kV, Korea Switching, Korea) and a function generator (FG-7002C, EZ Digital, Korea). To observe the characteristics of the applied electric voltage, we used a 1/1000 AC high-voltage reduction probe (P6015A, Tektronix, USA) and an oscilloscope (TDS2014, Tektronix, USA). In the experiments, the distance between spots and the droplet diameter were controlled by adjusting the speed of the stage translation apparatus from 0.5 to 2.0 mm/s. The flow rate of the syringe pump ranged from 3 μL/h to 5 μL/h. To determine the viability of patterned yeast cells, we incubated them at 25 °C as these conditions were known to be adequate for fermentation^26^.

For the alcoholic fermentation experiments, *Saccharomyces cerevisiae* (Korean Collection for Type Cultures, KCTC 7904) was used as the test yeast strain. We cultivated the *S. cerevisiae* cells in YPD (10 g/L Yeast extract, 20 g/L Peptone, 20 g/L Dextrose) medium at 30 °C. Stationary phase yeast cells were harvested through centrifugation (4000 × g, 15 min). We constructed an acrylic box (80 mm × 60 mm × 60 mm in size) that was internally separated by two partitions (thickness: 5 mm; height: 30 mm). Various yeast cell spreading conditions, such as the use of a fine-mist sprayer, non-patterned spreading, and patterned spreading with cell distances of 30, 100, and 1,000 μm were used in the fermentation experiments to analyze the effect of yeast cell arrangement on fermentation efficiency (Fig. 5b). Under all conditions, 1 mL of yeast cell medium (~10^6^ colony forming units, CFU/mL) was spread on a silicon wafer and incubated with 200 mL of squeezed grape solution (sugar content: 27.2 g/200 mL) at 25 °C for a maximum of 120 h in the acrylic box. The alcohol concentration in the tested solutions was measured using an alcolyzer (Anton Paar DMA 4500, Alcolyzer Wine, Austria). In the yeast cell patterned spreading conditions, the flow rate of yeast medium supplied to the system was 0.2 mL/h, the spray tip had an inner diameter of 100 μm, and the applied electricity was AC 3 kV. In addition, the patterned cell distances were controlled by adjusting the speed of the stage transition apparatus (0.5–8.5 mm/s).
